# Update to: A stakeholder workshop about modelled maps of key malaria indicator survey indicators in Madagascar

**DOI:** 10.1186/s12936-019-3052-z

**Published:** 2020-01-09

**Authors:** Rosalind E. Howes, Kaleem Hawa, Voahangy Fanomezana Andriamamonjy, Thierry Franchard, Raharizo Miarimbola, Sedera Aurélien Mioramalala, Jean Florent Rafamatanantsoa, Mirana Ando Mbolatiana Rahantamalala, Solo Harimalala Rajaobary, Hariniaina David Gaël Rajaonera, Andrianiaina Parfait Rakotonindrainy, Clairaut Rakotoson Andrianjatonavalona, Dina Ny Aina Liantsoa Randriamiarinjatovo, Faratiana Michèle Randrianasolo, Rado Malalatiana Ramasy Razafindratovo, Masiarivony Ravaoarimanga, Maurice Ye, Peter W. Gething, Cameron A. Taylor

**Affiliations:** 1 Malaria Atlas Project, Nuffield Department of Medicine, Big Data Institute, University of Oxford, Oxford, UK; 2 National Malaria Control Programme, Ministry of Health, Antananarivo, Madagascar; 3 Ministry of Health, Antananarivo, Madagascar; 4 Faculty of Science, University of Antananarivo, Antananarivo, Madagascar; 5 Department of Public Health, Faculty of Medicine, University of Antananarivo, Antananarivo, Madagascar; 6 Direction des Études et de la Planification, Ministry of Health, Antananarivo, Madagascar; 7 Direction du Système d’Information, Ministry of Health, Antananarivo, Madagascar; 8 Institut Pasteur de Madagascar, Antananarivo, Madagascar; 9 MEASURE-Evaluation, ICF, Antananarivo, Madagascar; 10 The DHS Program, ICF, Rockville, USA

Following publication of the original article [1], the authors expressed their wish to provide a French version of the article.

To this end, please find a (French) translation of the article in the additional file included with this update (Additional file 1).

By providing this translation, the authors hope to expand the readership of their article; for example, in French the article is more widely accessible to Malagasy people wanting to know more about the outcomes of the meeting detailed in the article.

The authors thank you for reading this update.

## Supplementary information

**Additional file 1.** French translation of meeting report.
