# Supplementary material for: Update to: A stakeholder workshop about modelled maps of key malaria indicator survey indicators in Madagascar
Source: Malar J. 2020 Jan 9;19:13. doi: 10.1186/s12936-019-3052-z (PMC6953134; doi:10.1186/s12936-019-3052-z)
Supplement: Supplementary file 1 — Additional file 1. French translation of meeting report. [file 12936_2019_3052_MOESM1_ESM.docx]

**Un atelier de parties prenantes sur les cartes modélisées des indicateurs-clés du paludisme à Madagascar**

Rosalind E. Howes^1^, Malaria, Kaleem Hawa^1^, Voahangy Fanomezana Andriamamonjy^2^, Thierry Franchard^3,4^, Raharizo Miarimbola^3,5^, Sedera Aurélien Mioramalala^3,5^, Jean Florent Rafamatanantsoa^6^, Mirana Ando Mbolatiana Rahantamalala^2,5^, Solo Harimalala Rajaobary^2^, Hariniaina David Gaël Rajaonera^2^, Andrianiaina Parfait Rakotonindrainy^7^, Clairaut Rakotoson Andrianjatonavalona^6^, Dina Ny Aina Liantsoa Randriamiarinjatovo^8^, Faratiana Michèle Randrianasolo^2^, Rado Malalatiana Ramasy Razafindratovo^3,5^, Masiarivony Ravaoarimanga^8^, Maurice Ye^9^, Peter W. Gething^1^, Cameron A. Taylor^10^

^1^Malaria Atlas Project, Nuffield Department of Medicine, Big Data Institute, University of Oxford, Oxford, UK

^2^Programme National de Contrôle du Paludisme, Ministère de la Santé, Antananarivo, Madagascar

^3^ Ministère de la Santé, Antananarivo, Madagascar

^4^Faculté des Sciences, Université d’Antananarivo, Antananarivo, Madagascar

^5^Departement de Santé Publique, Faculté de Médecine, Université d’Antananarivo, Antananarivo, Madagascar

^6^Direction des Études et de la Planification, Ministère de la Santé, Antananarivo, Madagascar

^7^Direction du Système d’Information, Ministère de la Santé, Antananarivo, Madagascar

^8^Institut Pasteur de Madagascar, Antananarivo, Madagascar

^9^MEASURE-Evaluation, ICF, Antananarivo, Madagascar

^10^The DHS Program, ICF, Rockville, USA

*Auteurs correspondants

REH: rosalind.howes@bdi.ox.ac.uk

CAT: cameron.taylor@icf.com

**Résumé**

Le Programme des Enquêtes Démographiques et de Santé (The DHS Program) a effectué trois Enquêtes sur les Indicateurs du Paludisme (EIP) à Madagascar. Un ensemble de 13 indicateurs du paludisme collectés au cours de ces enquêtes ont été retenus pour être cartographiés sous forme de surfaces continues en utilisant des méthodes géostatistiques basées sur un modèle. Les opportunités et les limites de ces résultats cartographiés ont été discutés au cours d’un atelier qui s’est tenu en juillet 2018 à Antananarivo, Madagascar, qui a réuni 15 représentants de différentes agences d’exécution impliquées dans la recherche et la mise en œuvre de politiques à Madagascar. Les participants ont évalué les résultats à partir des cartes, les utilisant pour développer des graphiques et des textes pour appuyer leurs travaux dans le contrôle du paludisme à Madagascar.

**Mots-clés**  Madagascar, The DHS Program, Enquêtes sur les Indicateurs du Paludisme, cartes des indicateurs du paludisme, Modélisation géostatistique, atelier de travail

**Contexte**

Le Programme des Enquêtes Démographiques et de Santé (The DHS Program) à Rockville, dans le Maryland USA, financé par USAID, a fourni une assistance technique pour la réalisation d’enquêtes auprès des ménages dans plus de 90 pays, contribuant ainsi à faire progresser les connaissances dans les domaines de la population et de la santé, en particulier les tendances démographiques. [1] Les Enquêtes sur les Indicateurs du Paludisme (EIP) de 2011, 2013 et 2016 sont les enquêtes représentatives au niveau national les plus récentes effectuées à Madagascar (EIP) [2-4]. À la suite de l’EIP 2016, une requête a été déposée pour examiner plus en détails les résultats de ces enquêtes et aider les participants à renforcer leurs capacités à utiliser les données pour la prise de décision concernant la mise en place de programme. Étant donné la diversité épidémiologique du paludisme à Madagascar et les problèmes d’accessibilité dans tout le pays [5-7], une collaboration avec Malaria Atlas Project (MAP) a été proposée pour créer une série de cartes de surface modélisées présentant les résultats pour 13 indicateurs-clés du paludisme à partir des EIP 2013 et 2016 [8]. Le groupe de recherche MAP collabore avec l’Organisation Mondiale de la Santé (OMS) pour la modélisation géospatiale des maladies qui utilise une approche cartographique basée sur des faits pour modéliser des cartes spatiales continues et quantifier les mesures, y compris des estimations démographiques de la prévalence du paludisme dans la population et de son impact clinique [9-11]. Au moment de la rédaction de cet article, des cartes d’indicateurs modélisées pour 32 enquêtes de pays différents sont gratuitement disponibles sur le site Web : the DHS Spatial Data Repository.

Les surfaces spatialement modélisées des données DHS sont obtenues à partir d’une combinaison de donnes DHS, accessibles au public, d’ensembles de données globales environnementales et de l’utilisation de méthodes géostatistiques standardisées pour permettre la comparabilité entre pays et faciliter la prise décision politique et programmatique. Bien que la création de ces surfaces ne soit pas nouvelle, leur incorporation comme élément dans un processus de décision plus formel n’est pas encore courante.

Pour contribuer à une prise de décision informée sur les politiques et programmes d’intervention futurs sur le paludisme à Madagascar, The DHS Program et MAP ont collaboré pour créer un programme d’ateliers de travail consacrés à l’étude des surfaces spatialement modélisées des enquêtes EIP de 2013 et 2016. Les objectifs de l’atelier étaient de présenter la valeur ajoutée de la modélisation spatiale en déduisant des indicateurs métriques plus robustes et de contribuer à l’intégration des cartes dans le suivi et l’évaluation des indicateurs du paludisme à Madagascar.

**Pourquoi cartographier les indicateurs MIS**

Une meilleure compréhension de la variation géographique et de l’inégalité en termes de santé, de bien-être économique et d’accès aux ressources dans les pays est un élément qui est devenu central dans la réalisation des objectifs de contrôle du paludisme. [12]. Les indicateurs du paludisme évalués au niveau national dissimulent souvent des inégalités importantes dans des zones administratives/géographiques plus petites, en particulier les populations rurales pauvres les moins bien représentées. Alors que la prévalence du paludisme diminue et que les financements internationaux pour le contrôle du paludisme se restreignent, la capacité de cibler des ressources limitées vers des groupes défavorisés devient un problème crucial [13, 14]. Dans le même temps, la réalisation des objectifs pour les principaux indicateurs du paludisme n’est pas achevée et il reste des lacunes à combler. [15]. Le suivi des inégalités pour cibler les interventions de contrôle et mesurer les progrès accomplis dans la réalisation des objectifs de santé et de développement nécessite une base de preuves fiables, détaillées et désagrégées.

Différentes approches permettent actuellement d’estimer les indicateurs du paludisme pour de petites unités géographiques. Ces approches comprennent (i) l’augmentation des tailles d’échantillon des enquêtes nationales auprès des ménages pour garantir la représentativité de l’échantillon des unités administratives plus petites, (ii) l’utilisation de données des systèmes d’information de routine des établissements de santé ou des communautés, et (iii) des estimations pour les petites zones à l’aide de cartes spatialement interpolées qui utilisent des techniques de modélisation et statistiques pour prédire les valeurs pour de petites unités géographiques. L’augmentation des tailles d’échantillon entraine des coûts supplémentaires, financiers et en termes de temps, coûts qui souvent difficiles à soutenir dans un environnement aux ressources de plus en plus restreintes. En outre, non seulement la qualité et la représentativité des données du système d’information de santé de routine n’est pas toujours fiable mais aussi les données ne sont pas facilement accessibles. Dans les années récentes, c’est la troisième approche de l’interpolation spatiale qui a attiré un intérêt croissant. [12, 16, 17].

Le paludisme est très diversifié à Madagascar, et requiert différentes combinaisons d’interventions dans les écozones épidémiologiques du pays [5, 18]. Ces zones sont stratifiées basées sur la durée et l’intensité de la transmission et des taux de diagnostic positifs des années précédentes. Le plan d’échantillonnage de l’EIP de Madagascar a été conçu pour produire des indicateurs résumés à échelles épidémiologiques et programmatiques (n=5), ainsi qu’au niveau national. Cependant, la mise en œuvre du Programme est gérée au niveau du district de santé (n=114), et les résultats bruts de l’EIP ne permettent pas de déterminer les progrès de l’indicateur à cette échelle. Les approches géostatistiques peuvent utiliser les résultats des EIP disponibles au niveau de la grappe couplées avec des covariables environnementales pour prédire les valeurs de l’indicateur pour toutes les régions du pays. Cet ensemble de données plus efficaces permet d’agréger des indicateurs à des échelles utiles à des fins programmatiques [8].

**Structure de l’atelier et objectifs**

L’objectif de l’atelier sur les Surfaces modélisées à Madagascar consistait à fournir une assistance au Programme National de Lutte contre le Paludisme (PNLP, ou “Direction de Lutte contre le Paludisme”) et à d’autres partenaires qui collectent des donnés sur le paludisme pour l’interprétation et l’application de cartes modélisées qui illustre la variété géographique des indicateurs du paludisme à Madagascar. Au nombre des objectifs spécifiques de l’atelier, on peut citer la formation sur (i) la compréhension et l’interprétation correcte des indicateurs standard EIP, (ii) la compréhension de la création des surfaces modélisées, de leurs limites et de leurs hypothèses inhérentes, (iii) l’interprétation exacte des surfaces modélisées et (iv) identifier les scénarios dans les cartes pour répondre aux questions-clés programmatiques.

En prévision de l’atelier, des membres importants du PNLP, du ministère de la Santé, la Direction des Études et de la Planification, la Direction du Système d’Information et l’Organisme de Recherche de l’Institut Pasteur de Madagascar ont été contactés pour qu’ils désignent des membres de leur personnel pour participer à l’atelier. Les participants devaient avoir une expérience d’un logiciel de Systèmes d’Informations Géographiques (SIG) et, l’analyse des données sur le paludisme devait faire partie de leur travail. Au total, 15 personnes ont participé à l’atelier qui s’est tenu pendant quatre jours en juillet 2018 à Antananarivo, Madagascar.

Les activités organisées pendant toute la durée de l’atelier ont été conçues pour englober une gamme de techniques d’apprentissage pour des adultes telles que des présentations PowerPoint interactives, un apprentissage mixte, des démonstrations guidées, des exercices pratiques et des activités en petits groupes. La fin de l’atelier a été consacrée aux présentations finales par chaque équipe des sujets/indicateurs (généralement classés comme suit : vecteur, gestion de cas et morbidité) comprenant une question d’ordre programmatique qu’ils souhaitaient aborder, l’audience de leur présentation, une introduction au problème à Madagascar, les indicateurs sélectionnés pour l’analyse, les cartes appropriées et les interprétations/recommandations issues des surfaces modélisées.

**Méthodologie de la cartographie**

Le domaine de la statistique spatiale développe continuellement des modèles toujours plus complexes et raffinés [12, 16]. Cependant, les approches très personnalisées limitent la comparabilité des résultats du modèle, à la fois dans le temps et entre les sites. Par contre, l’approche méthodologique pour la cartographie des indicateurs de The DHS Program a été délibérément conceptualisée pour générer des résultats normalisés reposant sur des ensembles de données globalement disponibles, permettant ainsi une comparabilité complète entre les pays et les années d’enquête [8]. À partir des résultats de l’EIP de Madagascar, un sous-ensemble de 13 indicateurs du paludisme ont été identifiés comme étant appropriés pour l’analyse spatiale [19], et ont été modélisés avant l’atelier (Tableau 1). Ces surfaces (comprenant à la fois les prévisions moyennes et les cartes d’incertitude associées avec des intervalles de confiance à 95 %) sont disponibles gratuitement sur le site The DHS Spatial Repository, ainsi que des rapports détaillés sur les méthodes de cartographie utilisées [17, 19-22]: <http://spatialdata.dhsprogram.com/modeled-surfaces/>. Globalement, des cartes modélisées d’indicateurs sélectionnés à partir de 32 enquêtes MIS/DHS de 31 pays sont actuellement disponibles.

Comme on l’a décrit précédemment, il s’est avéré qu’une approche géostatistique basée sur un modèle (MBG) a été considérée comme plus appropriée pour générer des sorties spatiales standardisées à partir des résultats de grappes EIP brutes [8, 20]. L’interpolation spatiale est le fondement de MBG. Les estimations de chaque cellule de la grille sont basées sur des observations d’enquêtes proches couplées à des modèles géographiques de surfaces de covariables biologiquement pertinentes et spatialement continues (Tableau 2). Le processus de modélisation caractérise les tendances observées dans les données d’enquête en quatre composantes : la variance d’échantillonnage est représentée par un modèle d’échantillonnage binomial; les erreurs non dues à l’échantillonnage sont expliquées par des effets fixes (par une relation de régression multivariée définie en reliant les variables des indicateurs aux covariables) et des effets aléatoires (processus de Gauss paramétré par une fonction de covariance spatiale de Matern); et enfin, un simple terme de bruit gaussien représente la variation résiduelle. Tous les paramètres du modèle sont estimés conjointement dans un cadre bayésien [8, 20] qui génère des prévisions au niveau des pixels pour chaque indicateur à travers le pays, en se basant sur les modèles des covariables environnementales pertinentes sélectionnées par le modèle pour leur corrélation spatiale avec les données brutes de l’indicateur.

Ces concepts méthodologiques globaux ont été présentés au cours de l’atelier, de même qu’une discussion sur les statistiques descriptives exploratoires de données spatiales (y compris des variogrammes et des histogrammes représentant la structure des valeurs de l’indicateur) et les statistiques de validation du modèle. L’objectif de l’atelier était de fournir un aperçu conceptuel des méthodes de cartographie et de permettre une évaluation critique appropriée des résultats modélisés. En tant que tel, un accent important a été également mis sur les limites des résultats (c’est-à-dire la faiblesse de la modélisation des zones urbaines et rurales et les difficultés de temporalité dans les mesures de l’indicateur [17]) et l’importance de l’évaluation la confiance relative dans les prévisions entre zones. Des exemples de manipulations à base de SIG des surfaces modélisées ont ensuite été présentées et testées par les participants.

**Mettre les cartes en pratique : études de cas exploratoires d’applications programmatique des cartes d’indicateurs MIS modélisées**

Les deux derniers jours de l'atelier ont surtout permis aux participants d'explorer les cartes modélisées pour en tirer des recommandations pertinentes sur le plan programmatique, recommandations qui pourraient être appliquées, de manière plausible, dans le contexte de leurs positions actuelles. Les résultats de ces discussions de groupe sont résumés ici. Ils illustrent différentes manières dont les surfaces modélisées des enquêtes de The DHS Program peuvent être appliquées facilement et rapidement par les PNLP et les autres parties prenantes. Des analyses plus formelles ont été encouragées, mais celles-ci se situaient en dehors du calendrier de l'atelier.

*Exemple 1: Renforcement de l’accès au traitement préventif intermittent pendant la grossesse*

Le traitement préventif intermittent de la Sulfadoxine-Pyriméthamine (SP) pendant la grossesse a été adopté à Madagascar en 2004, et son utilisation progressive a conduit à sa mise en œuvre dans tous les districts en phase de contrôle. En 2015, Madagascar a commencé à appliquer la recommandation de l'OMS consistant à augmenter le nombre minimum de doses de deux (TPIg2 +) à trois (TPIg3 +). Cela étant, l'indicateur de couverture TPIg3 + n'était pas approprié pour examiner compte tenu de la temporalité de sa définition (ayant pour dénominateur le « Nombre total de femmes interviewées ayant eu une naissance dans les deux années précédant l’enquête » [23], c'est-à-dire qu'elles étaient antérieures à la mise en œuvre locale de la politique TPIg3 +). On a préféré plutôt évaluer l’indicateur de couverture TPIg2+. L'agrégation au niveau des districts de la surface modélisée a indiqué que la couverture en 2016 est restée inférieure à 20 % dans 40 % des districts cibles (Fig. 1A). Ce résultat concordait avec une couverture relativement faible des consultations prénatales selon laquelle environ un tiers de femmes enceintes n'avait jamais effectué une consultation prénatale (source : Ministère de la Santé, 2017), et des ruptures régulières de stocks de SP, notifiées par les établissements de santé (43 % l'ont fait en 2016 ; source : PNLP, 2017).

Les surfaces modélisées ont mis en évidence un certain degré d’hétérogénéité spatiale dans la couverture du TPIg2+ (Fig. 1A), avec une incertitude prédictive élevée. (>20 %) également généralisée (Fig. 1B) probablement associée aux tailles d’échantillon relativement petites inhérentes à ces indicateurs (N=2 786, par rapport à 10 816 enquêtées pour d’autres indicateurs en 2016).

Des recommandations ont été formulées pour augmenter l’effort d’échantillonnage dans les districts côtiers à forte incertitude au cours des futures EIP, et pour renforcer la notification du TPIg au cours des consultations prénatales dans les établissements de santé. Il a été également recommandé de renforcer la collaboration entre le PNLP et le programme National de Santé Familiale, parallèlement à la relance de campagnes de sensibilisation ciblant les zones ayant les couvertures les plus faibles (Fig. 1A).

*Exemple 2 : Tendances spatio-temporelles de l’accès aux moustiquaires imprégnées d’insecticide et implications sur les futures campagnes de distribution de masse*

Madagascar vise la couverture universelle des moustiquaires imprégnées d’insecticide dans tous les districts en phase de contrôle. Cet objectif est réalisé principalement par le biais de campagnes de distribution de masse, les dernières ayant eu lieu en 2012-2013, 2015 et 2018 [18, 24]. L’objectif actuel est de permettre à au moins 90 % des ménages des districts cibles de disposer d’au moins une MII pour deux résidents. Plusieurs circuits de distribution continue complètent les campagnes de masse, notamment les consultations prénatales, les distributions par les agents de santé communautaires et les ventes subventionnées dans les communautés périurbaines. Les EIP de 2013 et 2016 ont donc évalué l'impact global de ces activités, permettant de mesurer l'évolution de la couverture au cours de cette période.

L’interprétation des résultats de l’enquête doit tenir compte du calendrier des campagnes de distribution de masse. L’EIP 2013 s’est déroulée à mi-parcours d’une campagne de distribution, 31 districts ayant été couverts au cours des six mois précédant l’EIP et 61districts après l’EIP. En revanche, tous les districts cibles ont été inclus dans la distribution de MII au cours des six mois précédant L’EIP 2016.

Plusieurs indicateurs de couverture en MII basés sur différents dénominateurs (ménages *vs.* résidents) ont été inclus dans les analyses spatiales, chacun représentant différents aspects de l’impact du programme. (Tableau 1). Ici, les participants ont sélectionné trois de ces indicateurs pour évaluer la couverture en MII à Madagascar en 2016 (Fig. 2A-C) et les changements relatifs du niveau de ces indicateurs depuis 2013 (Fig. 2D-F), pour savoir quelles leçons pourraient être tirées des surfaces modélisées pour les futures campagnes de distribution. Celles-ci comprenaient la portée spatiale des campagnes de distribution utilisant l’indicateur de présence de MII dans le ménage. (Fig. 2A et 2D), l’adéquation de la couverture en tenant compte du nombre de résidents du ménage (l’objectif est une moustiquaire pour deux personnes ; Fig. 2B et 2E), et enfin l’utilisation des moustiquaires disponibles. (Fig. 2C et 2F). Les surfaces cartographiées ont été agrégées par unités de district afin de refléter le niveau auquel la prise de décision et la logistique sont coordonnées pendant les campagnes MII.

Les surfaces modélisées ont montré que malgré un niveau assez élevé en couverture de MII, 52 des 92 districts ciblés ayant plus de 90 % des ménages possédant au moins une moustiquaire, la couverture avait néanmoins chuté quand on considère la disponibilité des MII en fonction de l’objectif d’une MII pour deux personnes par ménage. Dans aucun district, l’objectif national de 90 % en 2016 n’est atteint, bien que dans 39 districts (42 %) les niveaux étaient >75 %. Néanmoins, les indicateurs d’utilisation rapportés par les résidents des ménages indiquaient que les MII étaient utilisées à des taux suggérant que même quand leur nombre était insuffisant dans le ménage, les résidents dormaient sous les moustiquaires qui étaient disponibles. La couverture de ces indicateurs était généralement meilleure dans les zones côtières où la transmission est plus élevée [6], en particulier le long des districts Nord des côtes Ouest et Est. La couverture a chuté dans les zones montagneuses où la transmission est moins intense. Les cartes suggèrent des changements généralement soutenus ou positifs dans la couverture entre 2013 et 2016, les améliorations étant particulièrement importantes dans les districts du Sud, même si, au niveau national, la couverture dans ces districts reste parmi les plus faibles. Dans les districts de la côte Est, où la transmission est la plus élevée et où la couverture en MII est encore inférieure aux objectifs nationaux, on n’observe que peu de changements depuis 2013. Toutefois, les prévisions du modèle comportaient de grandes zones d'incertitude élevée, notamment dans les cartes d'accessibilité et de disponibilité des MII, où les estimations cartographiques doivent être interprétées avec prudence (Fig. 2B-C et 2E-F). L'hétérogénéité spatiale dans les résultats au niveau des grappes peut expliquer l'incertitude dans ces domaines.

Les recommandations pour les campagnes futures issues de ces cartes modélisées visaient principalement à augmenter le nombre de moustiquaires distribuées, en intensifiant les efforts, en particulier dans les zones de la côte Est où la couverture était faible malgré une transmission relativement élevée. Les résultats sur l’utilisation étaient encourageants mais toujours insuffisants, indiquant que d’autres interventions de communication du comportement seraient importantes parallèlement aux distributions, conformément aux directives du PNLP.

*Exemple 3 : Recherche de traitement pour les enfants avec de la fièvre*

La recherche d'un traitement pendant un épisode de fièvre est la première étape importante vers une gestion efficace des cas et une réduction de la morbidité due au paludisme, ainsi que vers la garantie d'un signalement fiable des épisodes de paludisme à des fins de surveillance. Les faibles taux de recherche de traitement dans une grande partie de l'Afrique sont l'une des principales raisons pour lesquelles l'OMS utilise des sources de données indépendantes de la surveillance de routine dans ses estimations de la charge de cas cliniques [15, 25]. L'indicateur MIS qui quantifie cela est le taux de recherche de traitement par la mère pour tout enfant de moins de cinq ans ayant eu de la fièvre au cours des deux semaines précédant l'enquête.

Les résultats de l’EIP au niveau national à Madagascar suggèrent une augmentation du taux de recherche de traitement, mais qui demeure néanmoins à un niveau faible, ce taux, tout prestataire de santé confondu, variant de 38 % en 2013 à 46 % en 2016. Seulement respectivement 29 % et 36 % ont recherché un traitement dans un centre de santé public susceptible de fournir une gestion de cas gratuite et appropriée et de rapporter des estimations de cas mensuelles à la base de données centralisée du ministère de la Santé. Ces faibles taux de contact avec les prestataires de soins de santé recommandés sont une cible du Plan stratégique national actuel de Madagascar par le biais d’activités de communication visant à modifier les comportements. Une meilleure compréhension des tendances de cet indicateur contribuerait à cibler les efforts futurs en fonction des lacunes actuelles et des niveaux de risque d’infection locaux.

L’indicateur de recherche de traitement MIS a donc été évalué pour déterminer quelles tendances spatio-temporelles pouvaient être dégagées au-delà des chiffres de synthèse nationaux. Des statistiques descriptives sont présentées pour 2013 (Fig. 3A-C) et 2016 (Fig. 3D-F). Les nombres bruts au niveau des grappes ont mis en évidence un niveau élevé d'hétérogénéité spatiale (figures 3A et 3D), probablement associé à des tailles d’échantillon différentes et parfois petites (n2013 = 633 et n2016 = 1096 mères éligibles dans l'ensemble du pays dont les enfants ont eu de la fièvre dans les deux semaines précédant l’enquête, qui deviennent très faibles lorsqu’on les considère au niveau des grappes). Les valeurs de points de données brutes (figures 3A et 3D) et les variogrammes (figures 3B et 3E) ont révélé que l'ensemble de données avait une structure spatiale limitée, reflétée également par une incertitude relative élevée dans les cartes prédites. De manière cohérente avec ces caractéristiques, les prédictions du modèle ont révélé une faible corrélation avec les données brutes observées au niveau des grappes (figures 3C et 3F). Ces avertissements dans les données suggèrent que les prévisions spatiales de cet ensemble de données et de ce modèle actuel ne sont peut-être pas fiables.

Ces informations indiquent aux parties prenantes souhaitant améliorer les taux de prise en charge appropriée des cas de paludisme que les données et les modèles examinés ici sont insuffisants pour permettre des évaluations significatives des niveaux actuels de recherche de traitement. Des efforts supplémentaires seront nécessaires pour renforcer les données fondées sur des preuves et permettre de comprendre les tendances infranationales de cet indicateur. La faible taille des échantillons associée à cet indicateur MIS spécifique - en raison de sa nature opportuniste - limite la puissance statistique requise pour les analyses à haute résolution.

Des approches alternatives, telles que la détection active des cas, ou simplement des échantillons de taille plus grande, pourraient fournir des informations plus fiables sur cet indicateur important. Un message fort apparaît donc pour plaider en faveur du renforcement de cet indicateur, afin de permettre de répondre à des questions importantes sur la variabilité du comportement sur l'île et sur l'impact des initiatives du PNLP sur le changement de comportement dans le temps. Cela reste essentiel pour améliorer les taux de recherche de traitement et une gestion appropriée des cas, pierre angulaire de tout programme de contrôle.

**Évaluation des participants**

Les entretiens qualitatifs menés avant l’atelier avec le personnel du PNLP ont suggéré que la connaissance et l’utilisation des cartes par le programme se limitaient généralement à la cartographie descriptive des taux d’incidence globalement déclarés et aux résumés des grappes épidémiques. Les cartes modélisées des risques de paludisme n'ont pas été utilisées à des fins programmatique, ni aucune connaissance significative de ce qu'elles représentent.

Avant et après l’atelier, on a posé aux participants un test comprenant 13 questions pour évaluer leur connaissance des indicateurs MIS, des surfaces modélisées et de l’incertitude statistique. Le score moyen des participants est passé de 54 % à 87 %, tous les participants, sauf un, ayant amélioré leur performance (le score de ce participant étant demeuré constant à 86 %). Des formulaires d’évaluation anonymes ont également été remplis le dernier jour de l’atelier, donnant aux participants l’opportunité d’évaluer la pertinence, le rythme et le contenu de l’atelier et de faire des commentaires ou des suggestions. Les commentaires se sont révélés positifs. Les participants ont indiqué qu’ils avaient beaucoup appris des conférences et des exercices. Ils ont apprécié l’acquisition de connaissances et de compétences au cours de l’atelier et ils ont prévu d’intégrer les surfaces modélisées dans le cadre de la prise de décision à des fins programmatique dans le futur.

Tout au long de l'atelier, les participants ont identifié des exemples d’application de cartes modélisées qui pourraient être utilisées dans leurs domaines de travail spécifiques. On peut citer, par exemple, une prise de décision plus précise en l’absence de données complètes, une adaptation appropriée des interventions lors des épidémies à l'aide d'informations d'indicateurs spécifiques à une région donnée, une plus grande disponibilité d'informations dans des contextes aux ressources limitées, où des données supplémentaires sont difficiles à demander et l’utilisation comme outils de plaidoyer lors de la communication avec un public non spécialisé ou pour la synthèse d'informations dans les demandes de subvention. Le lancement imminent de la campagne de distribution de moustiquaires en 2018 a fourni une étude de cas très explicite sur la manière dont les cartes de surface modélisées pourraient aider à affiner les activités de planification futures, les cartes soulignant les zones pour lesquelles des ressources renforcées sont indispensables.

La critique la plus récurrente était que l'atelier n'était pas assez long. En particulier, la majorité des participants auraient souhaité bénéficier de plus de temps pour s'exercer à manipuler les cartes dans ArcGIS, ainsi que d'une formation au codage R. L'atelier a permis aux participants de discuter de la prise de décision basée sur des données à Madagascar et de nombreux observateurs ont déclaré que l'atelier leur avait permis de mieux comprendre les indicateurs du paludisme qu'ils jugeaient essentiels pour la prise de décisions au niveau du programme. Les discussions sur le contexte, les forces et les faiblesses des indicateurs, ainsi que sur la conception des études MIS, se sont révélées être un atout très précieux pour l’atelier

**Conclusions**

Il s'agissait du premier atelier sur les surfaces modélisées spécifique à un pays à être mis en œuvre par The Program DHS. Bien que ce type d’atelier ne soit pas recommandé pour tous les pays, il a été très bénéfique pour Madagascar, où plusieurs enquêtes EIP ont été réalisées et où le profil épidémiologie du paludisme est variable dans le pays. L’élaboration d’une gamme de cartes d’indicateurs modélisées et la formation des participants à l’atelier pour les évaluer de manière critique augmentent la capacité des parties prenantes de la lutte antipaludique dans le pays à prendre des décisions basées sur des données.

Celles-ci sont accessibles gratuitement sur le site Web du DHS Spatial Data Repository [21, 22], avec des cartes d'indicateurs sélectionnés à partir d'enquêtes EIP/DHS réalisées dans 30 autres pays. Tous les participants ont recommandé l'atelier à d'autres PNLP et certains ont demandé qu'une formation supplémentaire soit menée à Madagascar.

**Sigles**

DHS Enquête Démographique et de Santé

EIP Enquête sur les indicateurs du Paludisme

GIS Système d’Information Géographique

MAP Malaria Atlas Project

MBG Model-based Geostatistics

MII Moustiquaire Imprégnée d’Insecticide

MIS Malaria Indicator Survey

OMS Organisation Mondiale de la Santé

PNLP Programme National de Lutte contre le Paludisme

SP Sulfadoxine-Pyriméthamine

TPIg Traitement Préventif Intermittent pendant la grossesse

**Références**

1. The Demographic and Health Surveys (DHS) Program (https://dhsprogram.com/). Accessed: 28th August 2018

2. Institut National de la Statistique (INSTAT), Programme National de Lutte contre le Paludisme (PNLP), and ICF International. Madagascar Malaria Indicator Survey 2011 [Enquête sur les Indicateurs du Paludisme (EIPM)]. Calverton, MC, USA: INSTAT, PNLP, and ICF International (2011).

3. Institut National de la Statistique (INSTAT), Programme National de Lutte contre le Paludisme (PNLP), Institut Pasteur de Madagascar (IPM), and ICF International. Madagascar Malaria Indicator Survey 2013 [Enquête sur les Indicateurs du Paludisme (EIPM)]. Calverton, MC, USA: INSTAT, PNLP, IPM and ICF International (2013).

4. Institut National de la Statistique (INSTAT), Programme National de Lutte contre le Paludisme (PNLP), Institut Pasteur de Madagascar (IPM), and ICF International. Madagascar Malaria Indicator Survey 2016 [Enquête sur les Indicateurs du Paludisme (EIPM)]. Calverton, MC, USA: INSTAT, PNLP, IPM and ICF International (2016).

5. Howes RE, Mioramalala SA, Ramiranirina B, Franchard T, Rakotorahalahy AJ, Bisanzio D, et al. Contemporary epidemiological overview of malaria in Madagascar: operational utility of reported routine case data for malaria control planning. Malar J. 2016;15:502.

6. Kang SY, Battle KE, Gibson HS, Ratsimbasoa A, Randrianarivelojosia M, Ramboarina S, et al. Spatio-temporal mapping of Madagascar’s Malaria Indicator Survey results to assess *Plasmodium falciparum* endemicity trends between 2011 and 2016. BMC Med. 2018;16:71.

7. Ihantamalala FA, Herbreteau V, Rakotoarimanana FMJ, Rakotondramanga JM, Cauchemez S, Rahoilijaona B, et al. Estimating sources and sinks of malaria parasites in Madagascar. Nat Commun. 2018;9:3897.

8. Burgert-Brucker CR, Dontamsetti T, Gething PW. The DHS Program's Modeled Surfaces Spatial Datasets. Stud Fam Plann. 2018;49:87-92.

9. Hay SI, Snow RW. The Malaria Atlas Project: developing global maps of malaria risk. PLoS Med. 2006;3:e473.

10. Malaria Atlas Project (MAP) ([www.map.ox.ac.uk)](http://www.map.ox.ac.uk)). Accessed: 28th August 2018

11. Pfeffer DA, Lucas TCD, May D, Harris J, Rozier J, Twohig KA, et al. malariaAtlas: an R interface to global malariometric data hosted by the Malaria Atlas Project. Malar J. 2018;17:352.

12. Cohen JM, Le Menach A, Pothin E, Eisele TP, Gething PW, Eckhoff PA, et al. Mapping multiple components of malaria risk for improved targeting of elimination interventions. Malar J. 2017;16:459.

13. Drake TL, Lubell Y. Malaria and Economic Evaluation Methods: Challenges and Opportunities. Appl Health Econ Health Policy. 2017;15:291-97.

14. Drake TL, Lubell Y, Kyaw SS, Devine A, Kyaw MP, Day NPJ, et al. Geographic Resource Allocation Based on Cost Effectiveness: An Application to Malaria Policy. Appl Health Econ Health Policy. 2017;15:299-306.

15. WHO. World Malaria Report 2018. Geneva, Switzerland: World Health Organization (2018).

16. Dalrymple U, Mappin B, Gething PW. Malaria mapping: understanding the global endemicity of falciparum and vivax malaria. BMC Med. 2015;13:140.

17. Burgert-Brucker CR, Dontamsetti T, Marshall AMJ, Gething PW. Guidance for Use of the The DHS Program Modeled Map Surfaces. In DHS Spatial Analysis Reports No 14. Rockville, Maryland, USA: ICF International (2016). (https://dhsprogram.com/pubs/pdf/SAR14/SAR14.pdf)

18. National Malaria Control Programme of Madagascar. National strategic plan for malaria control in Madagascar 2018-2022. Progressive malaria elimination from Madagascar. (2017).

19. Burgert CR. Spatial interpolation with Demographic and Health Survey data: Key considerations. In DHS Spatial Analysis Reports No 9. Rockville, Maryland, USA: ICF International (2014). (<http://dhsprogram.com/pubs/pdf/SAR9/SAR9.pdf>)

20. Gething PW, Tatem AJ, Bird TJ, Burgert-Brucker CR. Creating Spatial Interpolation Surfaces with DHS Data. In DHS Spatial Analysis Reports No 11. Rockville, Maryland, USA: ICF International (2015). (https://dhsprogram.com/pubs/pdf/SAR11/SAR11.pdf)

21. Modeled Surfaces from the Madagascar 2013 Malaria Indicator Survey (https://spatialdata.dhsprogram.com/modeled-surfaces/#survey=MD|2013|MIS). Accessed: 28th August 2018

22. Modeled Surfaces from the Madagascar 2016 Malaria Indicator Survey (https://spatialdata.dhsprogram.com/modeled-surfaces/#survey=MD|2016|MIS). Accessed: 28th August 2018

23. MEASURE Evaluation, The Demographic and Health Surveys Program, President's Malaria Initiative, Roll Back Malaria Partnership, United Nations Children's Fund, World Health Organization. Household survey indicators for malaria control. (2018). (<http://www.malariasurveys.org/documents/Household%20Survey%20Indicators%20for%20Malaria%20Control_FINAL.pdf>)

24. President's Malaria Initiative. Madagascar Malaria Operational Plan Financial Year 2018. (2018). (https://[www.pmi.gov/docs/default-source/default-document-library/malaria-operational-plans/fy-2018/fy-2018-madagascar-malaria-operational-plan.pdf?sfvrsn=5](http://www.pmi.gov/docs/default-source/default-document-library/malaria-operational-plans/fy-2018/fy-2018-madagascar-malaria-operational-plan.pdf?sfvrsn=5))

25. Cibulskis RE, Aregawi M, Williams R, Otten M, Dye C. Worldwide incidence of malaria in 2009: estimates, time trends, and a critique of methods. PLoS Med. 2011;8:e1001142.

**Légendes des figures**

**Fig. 1 Couverture en 2016 des femmes ayant eu une naissance vivante au cours des deux années avant l’enquête qui ont reçu au moins deux doses prophylactique de SP/Fansidar (IPTp2+).** La carte spatialement continue est résumée au niveau du district (valeurs moyennes de l’indicateur ML_IPTP_W_2SA) dans le Panneau A, l’incertitude au niveau des pixels étant indiquée dans le Panneau B.

**Fig. 2 Caractéristiques de la couverture et de l’utilisation des MII au niveau du district pour 2016 (Panneaux A-C) et changement relatif par rapport à 2013 (Panneaux D-F)**. Les panneaux A et D correspondent au pourcentage de ménages avec au moins une MII (indicateur ML_NETP_H_ITN). Les panneaux B et E quantifient la proportion de la population ayant accès à une MII dans leur ménage quand elle est partagée par deux personnes au plus (ML_ITNA_P_ACC). Les panneaux C et F indiquent le pourcentage de résidents du ménage qui auraient dormi sous une MII la nuit précédant l'interview (ML_NETU_P_ITN). Les cartes spatialement continues sont agrégées au niveau du district et présentées sous forme de valeurs moyennes, avec une incertitude relative basée sur les moyennes brutes de la métrique d'incertitude au niveau du pixel

**Fig. 3 Statistiques descriptives des caractéristiques spatiales des taux de recherche de traitement par les mères d’enfants ayant eu de la fièvre dans les deux semaines ayant précédé l’interview (indicateur ML_FEVT_C_ADV).** Les panneaux AC représentent les données MIS de 2013 et les panneaux FD, celles de 2016. Les cartes des panneaux A et D illustrent les taux de recherche de traitement brut au niveau de la grappe, tandis que les panneaux B et E sont des variogrammes (une structure spatiale étroite montrerait un décalage croissant - ou dissimilarité entre les points - avec une distance spatiale croissante) Les panneaux C et F représentent la validation du modèle des valeurs brutes observées au niveau de la grappe (axe des ordonnées) par rapport aux valeurs prédites dans ces zones.

Lors de la validation du modèle, 25 % de l’ensemble des données sont retenus au hasard et le modèle est exécuté avec les 75 % restants. Ce processus est répété quatre fois sans remplacement, donnant ainsi des prévisions de validation pour tous les emplacements de grappe (panneaux C et F). Une corrélation étroite du diagramme de dispersion suggère une plus grande précision de la performance prédictive du modèle.

**Tableaux**

**Tableau 1. Indicateurs MIS des enquêtes réalisées à Madagascar en 2013 et 2016 sélectionnés pour la modélisation spatiale.** Les surfaces continues modélisées ainsi que des cartes d’intervalles crédibles à 95 % sont disponibles pour les deux années d’enquête sous forme d’images .png et de fichiers raster.tif à partir du référentiels de The DHS Program Spatial Data Repository [21, 22].

| **Indicateur** | **Définition** |
| --- | --- |
| MLFEVTCACT | Parmi les enfants de moins de cinq ans ayant eu de la fièvre au cours des deux semaines avant l’interview, pourcentage ayant pris une combinaison avec de l’artémisinine |
| MLFEVTCADV | Parmi les enfants de moins de cinq ans ayant eu de la fièvre au cours des deux semaines avant l’interview, pourcentage pour lesquels un traitement ou des conseils ont été recherchés |
| MLFEVTCBLD | Parmi les enfants de moins de cinq ans ayant eu de la fièvre au cours des deux semaines avant l’interview, pourcentage à qui on a prélevé du sang au doigt ou au talon pour être testé. |
| MLHEMOCHL8 | Pourcentage d’enfants de 6-59 mois dont le niveau d’hémoglobine est inférieur à 8,0 g/dl |
| MLIPTPW2SA | Pourcentage de femmes de 15-49 ans ayant eu une naissance vivante au cours des deux années avant l’enquête qui ont pris au cours de la grossesse deux doses ou plus de SP/Fansidar, dont au moins une dose au cours d’une visite prénatale (Traitement Préventif Intermittent pour les femmes enceintes (TPIg2+)) |
| MLIRSMHIRS | Pourcentage de ménages ayant bénéficié d’une pulvérisation d’insecticide intradomiciliaire (PID) au cours des 12 mois avant l’interview |
| MLITNAPACC | Pourcentage de la population (de fait) des ménages qui aurait pu dormir sous une moustiquaire imprégnée d’insecticide (MII) si chaque MII du ménage était utilisée par deux personnes ou plus. |
| MLNETCCITN | Pourcentage d’enfants de moins de cinq ans ayant dormi sous une MII la nuit avant l’interview |
| MLNETPHITN | Pourcentage de ménages avec au moins une MII |
| MLNETUPITN | Pourcentage de la population (de fait) des ménages qui ont dormi sous une MII la nuit avant l’interview |
| MLNETWWITN | Pourcentage de femmes enceintes qui ont dormi sous une MII la nuit avant l’interview |
| MLPMALCMSY | Pourcentage d’enfants de 6-59 mois présentant une infection paludéenne détectée par microscopie |
| MLPMALCRDT | Pourcentage d’enfants de 6-59 mois présentant une infection paludéenne détectée par un test de diagnostic rapide (TDR |

**Tableau 2. Cartographie des covariables utilisées dans la modélisation** [17].

| Nom court | Description | Source de données originales | Temporalité | Date |
| --- | --- | --- | --- | --- |
| Population | | | | |
| Access | Temps de voyage vers villes avec population > 50 000 via tous moyens de transport | http://forobs.jrc.ec.europa.eu | Statique | 2000 |
| NTL | Lumières de nuit VIIRS −2012 | http://ngdc.noaa.gov/eog/ | Statique | 2012 |
| GPW | Gridded Population of the World (GPW) densité de population | http://sedac.ciesin.columbia.edu/ | Statique | 2010 |
| Terre physique | |  |  |  |
| Elévation | Shuttle Radar Topography Mission (SRTM) Near−modèles d’élévation numérique quasi globaux (DEMs) | http://webmap.ornl.gov/ | Statique | 2000 |
| Environnement | |  |  |  |
| Aridité | Aridité annuelle moyenne | http://csi.cgiar.org/Aridity/ | Synoptique | 1950−2000 |
| PRECIP | Moyenne des precipitations mensuelles | http://www.worldclim.org/ | Synoptique | 1950−2000 |
| EVI | Indice de végétation amélioré | http://modis.gsfc.nasa.gov/ | Multitem-porel | 2001−2014 |
| LST.day | température de la surface du sol pendant la journée | http://modis.gsfc.nasa.gov/ | Multitem-porel | 2001−2014 |
| LST.delta | Plage de fluctuation quotidienne de la température de surface du sol pendant la journée | http://modis.gsfc.nasa.gov/ | Multitem-porel | 2001−2014 |
| LST.night | Plage de fluctuation quotidienne de la température de surface du sol pendant la nuit | http://modis.gsfc.nasa.gov/ | Multitem-porel | 2001−2014 |
| PET | Évapotranspiration potentielle annuelle moyenne | http://csi.cgiar.org/Aridity/ | Synoptique | 1950−2000 |
| TCB | Luminosité de Tasseled−cap | http://modis.gsfc.nasa.gov/ | Multitem-porel | 2001−2014 |
| TCW | Humidité de Tasseled−cap | http://modis.gsfc.nasa.gov/ | Multitem-porel | 2001−2014 |


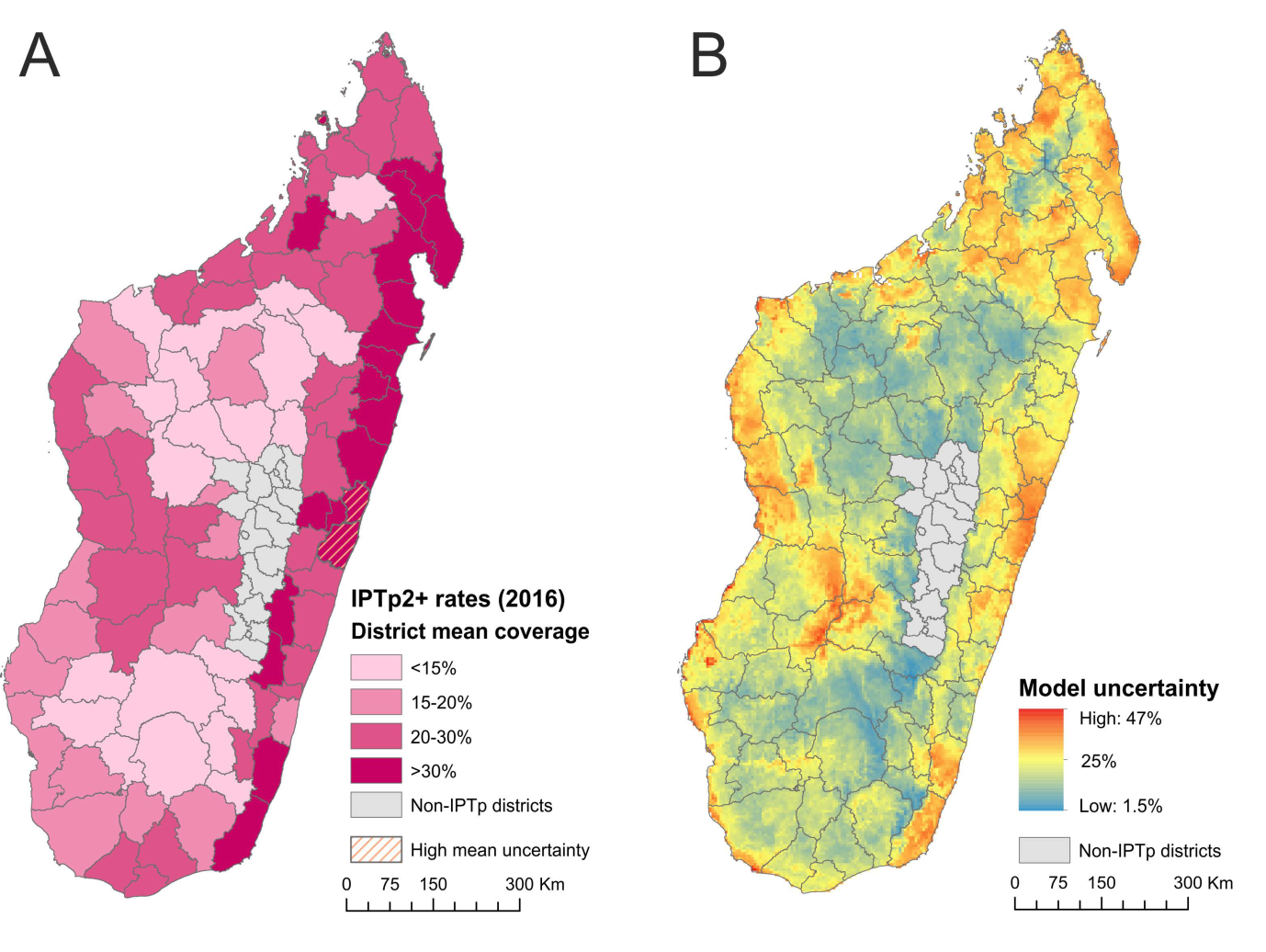


Figure 1


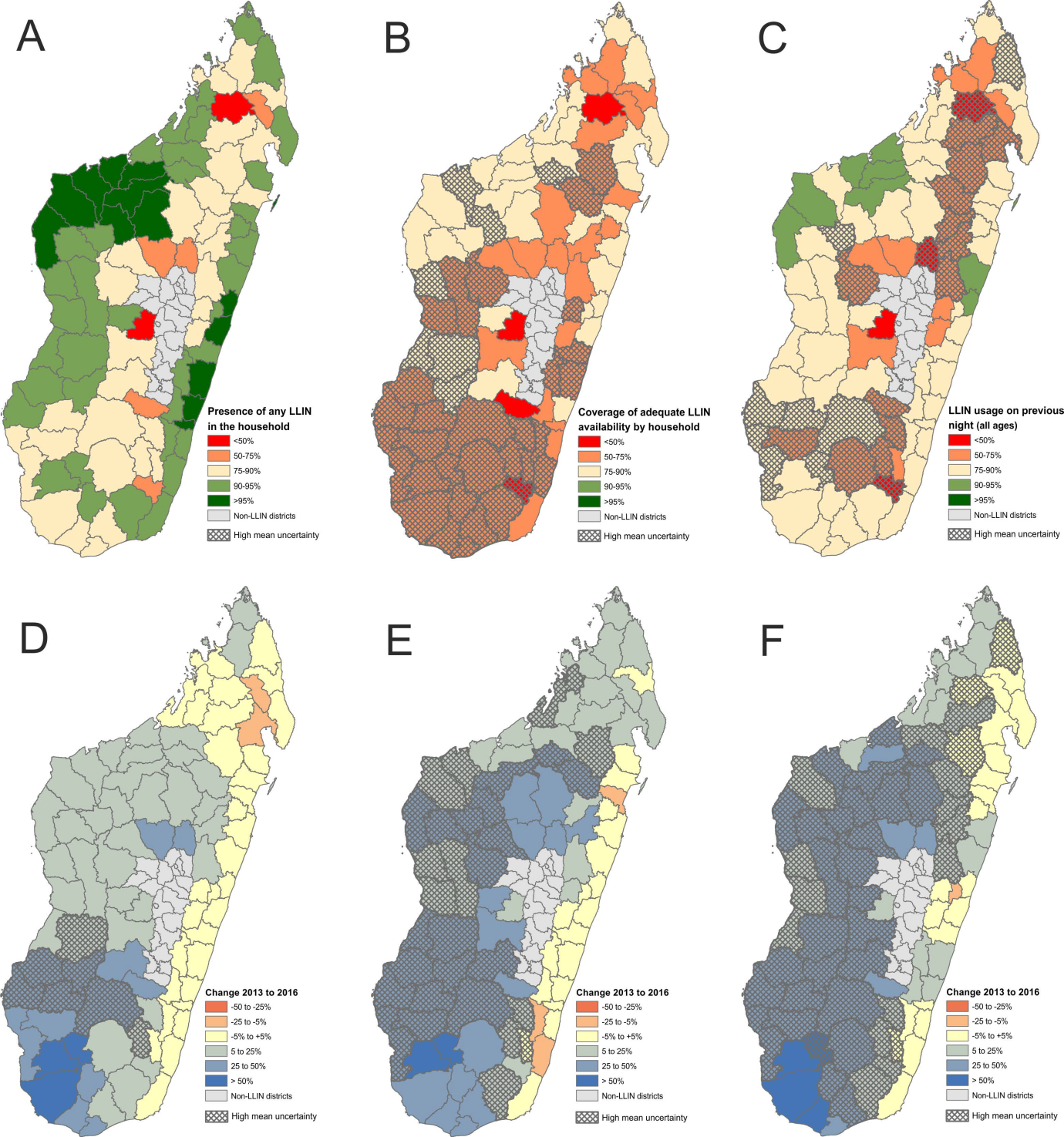


Figure 2


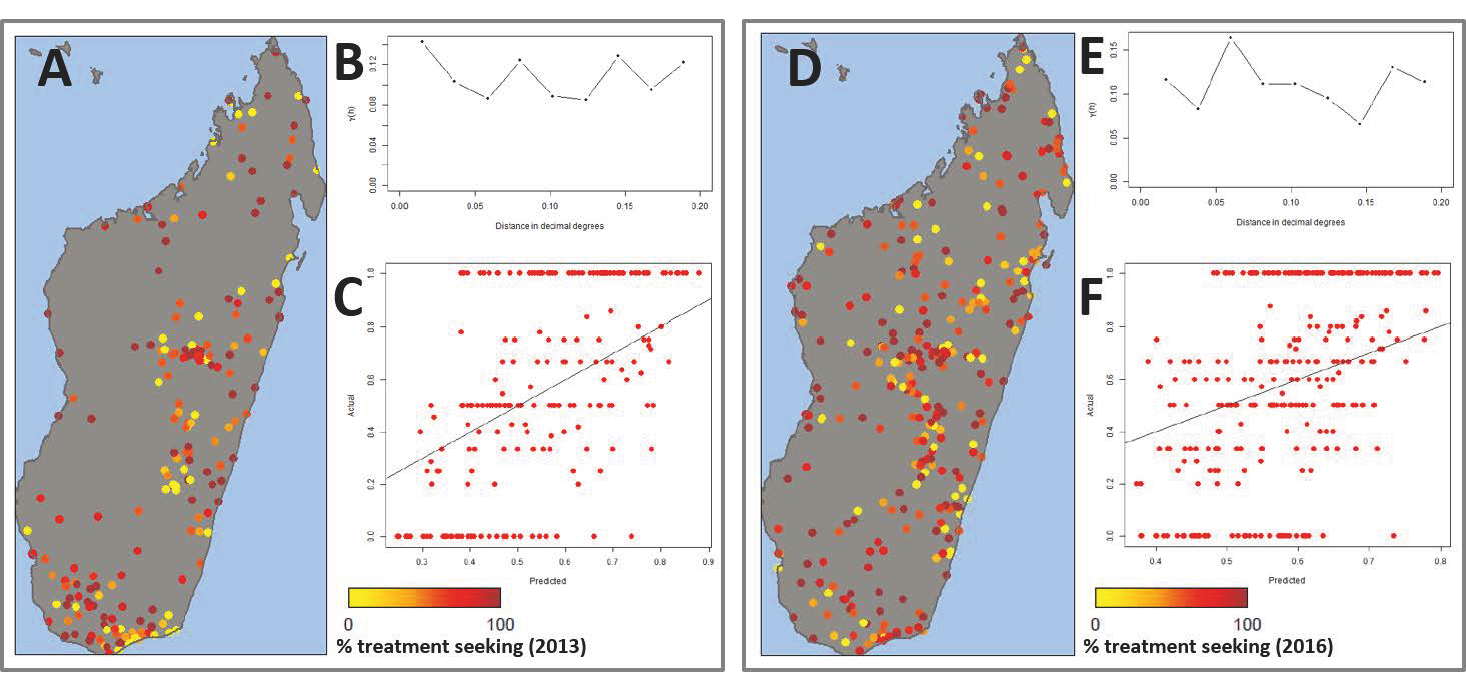


Figure 3
